# Exploring effective approaches for haplotype block phasing

**DOI:** 10.1186/s12859-019-3095-8

**Published:** 2019-10-30

**Authors:** Ziad Al Bkhetan, Justin Zobel, Adam Kowalczyk, Karin Verspoor, Benjamin Goudey

**Affiliations:** 1 School of Computing & Information Systems, University of Melbourne, Parkville, 3010 Australia; 2 Centre for Neural Engineering, University of Melbourne, Carlton, 3053 Australia; 3 Faculty of Mathematics and Information Science, Warsaw University of Technology, Warsaw, 00-662 Poland; 4 Centre for Epidemiology and Biostatistics, The University of Melbourne, Parkville, 3010 Australia; 5 IBM Australia - Research, Southgate, 3006 Australia

**Keywords:** Haplotype estimation, Phasing, Haplotype blocks, Haplotype analysis

## Abstract

**Background:**

Knowledge of phase, the specific allele sequence on each copy of homologous chromosomes, is increasingly recognized as critical for detecting certain classes of disease-associated mutations. One approach for detecting such mutations is through phased haplotype association analysis. While the accuracy of methods for phasing genotype data has been widely explored, there has been little attention given to phasing accuracy at haplotype block scale. Understanding the combined impact of the accuracy of phasing tool and the method used to determine haplotype blocks on the error rate within the determined blocks is essential to conduct accurate haplotype analyses.

**Results:**

We present a systematic study exploring the relationship between seven widely used phasing methods and two common methods for determining haplotype blocks. The evaluation focuses on the number of haplotype blocks that are incorrectly phased. Insights from these results are used to develop a haplotype estimator based on a consensus of three tools. The consensus estimator achieved the most accurate phasing in all applied tests. Individually, EAGLE2, BEAGLE and SHAPEIT2 alternate in being the best performing tool in different scenarios. Determining haplotype blocks based on linkage disequilibrium leads to more correctly phased blocks compared to a sliding window approach. We find that there is little difference between phasing sections of a genome (e.g. a gene) compared to phasing entire chromosomes. Finally, we show that the location of phasing error varies when the tools are applied to the same data several times, a finding that could be important for downstream analyses.

**Conclusions:**

The choice of phasing and block determination algorithms and their interaction impacts the accuracy of phased haplotype blocks. This work provides guidance and evidence for the different design choices needed for analyses using haplotype blocks. The study highlights a number of issues that may have limited the replicability of previous haplotype analysis.

## Background

Most genetic studies focus on analyzing genotypes to detect significant genetic associations with diseases [1]. However, it has long been recognized that some disease-associated haplotypes, the specific allele sequence on each copy of homologous chromosomes, may be undetectable with a focus on genotype alone [2, 3]. For example, the different allocation of specific alleles on each copy of a chromosome pair (which is ignored by genotype analysis) can impact the gene expression of an associated gene in a different manner [2]. Phasing, also known as haplotype estimation, reconstructs the haplotype sequences from genotype data and has been essential for understanding sequence-specific variation such as allele-specific expression [4, 5], methylation effects [6], and compound heterozygosity [2, 7]. Moreover, numerous studies have shown that haplotype-based association analysis can identify variants that would be missed by a standard single nucleotide polymorphism (SNP)-based analysis [8–10].

**Fig. 1:**
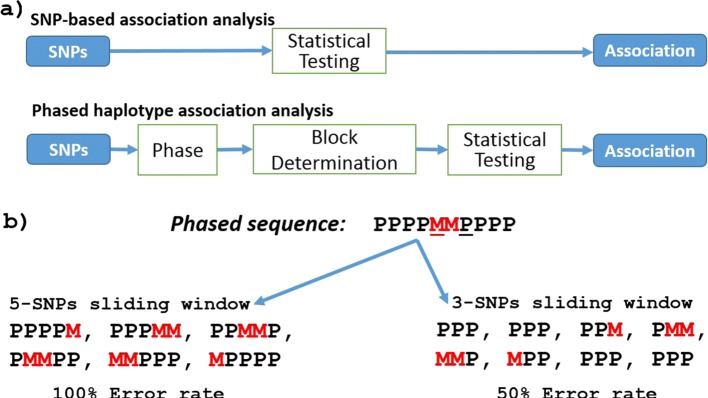
The relationship between phasing and block determination in haplotype association analysis: **a** Outline of the core steps within a standard SNP-based and a haplotype association analysis, highlighting the additional complexity of the haplotype-based approach. **b** the effect of block determination given a phased sequence. Here, we show a haplotype from 10 heterozygous SNPs. While all the alleles should all be allocated to the same allele, 2 have been allocated to the maternal v (marked as M in red) and the remaining to the paternal sequence (marked as P in black). As such, the phased sequence has 2 switches (phasing errors) at the loci 5 and 7 and hence has a switch error of 2/10 (20%). If we chose a 3-SNP sliding window with one SNP step, we find that 4/8 (50%) of the blocks contain errors. However, if a 5-SNP window was used, all blocks will contain errors. Using different window sizes affect the error rate within the blocks, yet switch error calculated at the whole sequence scale is constant 20%

Despite its promise, phased haplotype association analysis is not commonly applied in genome-wide association studies, likely due to the increased complexity of haplotype analysis. As shown in Fig. 1a, the analysis requires three main steps: phasing, block determination and statistical analysis. A wide range of algorithms have been designed for phasing of the genome, with recent algorithms scaling to hundreds of thousands of individuals [11]. Block determination, overlapping or non-overlapping, typically uses a fixed-size sliding window [9] or is based on linkage disequilibrium (LD) so that each block contains alleles that are more likely to be inherited together [12, 13]. Finally, the phased blocks are assessed statistically to determine significant association with disease.

In haplotype association analysis, replicability of detected associations will be affected by the error rate within the phased haplotype blocks, which is dependent on both the error rate of the chosen phasing method and the approach chosen for block determination. An example of how block determination methods impact error rates is shown in Fig. 1b, where the phased sequence contains switch errors (i.e. it swaps between paternal and maternal allocations) at loci 5 and 7. However, the proportion of haplotypes blocks that contain phasing errors varies from 50% to 100% depending on whether a 3- or 5-SNP sliding window (shifted by one SNP at each step) approach is used. This simple example shows how both the initial phasing accuracy and the blocks determination method selected can impact the accuracy of haplotypes within a set of determined blocks.

Current haplotype association studies assume that haplotypes within the determined blocks (either LD or sliding window based) do not contain errors due to phasing [9, 12, 13]. This assumption may increase false positive associations [3, 14] given that phased haplotypes tend to be accurate only in short regions and this accuracy cannot be maintained for long regions [15, 16]. Some genomic regions contain more errors than others due to factors such as differences in linkage disequilibrium, recombination rate, and the density of SNPs [17]. The impact of this assumption on downstream analysis is unclear as the error rate of phased haplotype blocks has not been explored.

Existing evaluations of phasing tools have been conducted without considering the intended application of the phased haplotypes. Such studies evaluate phasing methods using metrics such as switch error, missing error, incorrect genotype percentage, and performance time [15, 16, 18, 19]. While these metrics are informative, they are typically reported as aggregates from across either a set of genomes or a single individual’s entire genome. Such summary statistics do not necessarily reflect the quality of the phased haplotypes within specific regions or blocks, which is critical for downstream haplotype block analysis. Furthermore, these evaluations do not consider the downstream application of phased haplotypes and hence do not consider the joint impact of chosen phasing and block determination methods on phasing error rate of resulting haplotype blocks.

In this paper, we present the first evaluation of the behaviour of state-of-the-art phasing tools, as it relates to the direct use of phased haplotype blocks in downstream association analysis. We evaluate seven well-known population-based haplotype estimation methods (fastPHASE, BEAGLE, IMPUTE, MaCH, SHAPEIT2, HAPI-UR, and EAGLE2) in addition to consensus haplotype estimators, which combine results from multiple phasing tools. We examine the interaction between phasing tool and two block determination approaches, based on sliding windows and LD thresholds, and their joint impact on error rates of derived haplotype blocks. We consider two different scenarios when phasing a particular region; either phasing the entire chromosome and then extracting the region, or extracting the region and then conducting phasing. Finally, the stability of the tools when applied to the same datasets several times is reported for all evaluation metrics.

## Results

### Different error locations obtained by different phasing tools

**Fig. 2:**
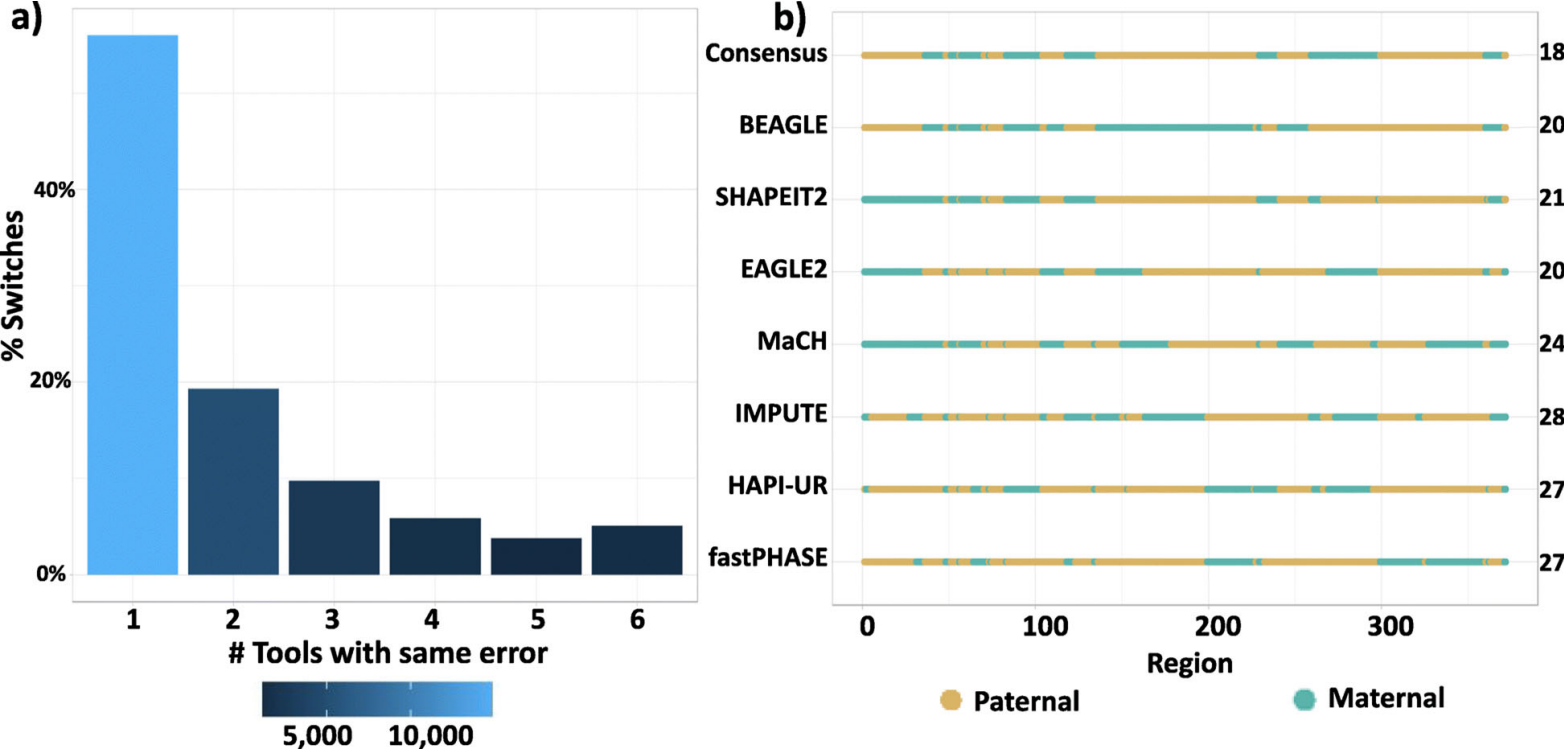
Similarity of switch location across phasing tools: **a** The frequency of shared switch error sites by different numbers of tools. The first bar represents the frequency of the error sites reported by one tool. The second bar represents the frequency of the error sites reported by two tools, and so on. **b** Estimated haplotypes for a random individual from seven different tools. The shown sequence corresponds to 372 heterozygous SNPs out of 1400 SNPs in the region Chr17:11045667-17395608. Each line represents a haplotype estimated by the corresponding tool, with the complementary phasing not shown (paternal regions become maternal and vice versa). Green represents the correctly phased haplotype runs that are identical to the maternal haplotype, while the brown runs are identical to the paternal ones. The numbers aligned to each tool name are the count of switches between the paternal and maternal copies

The switch errors observed across the six tools in chromosome 1 occurred at 12,145 different loci out of a possible 36,923 heterozygous SNPs. The distribution of error similarity by tool shown in Fig. 2a. The majority of these switches were unique to a single tool (∼ 56*%*), while only ∼ 5*%* of these loci were common between all tools. This large variation between tools in the sites of the switch errors implies that most tools are likely to result in different haplotypes being formed and hence may have a strong impact on haplotype analysis.

Examining the phasing of individual chromosomes highlights the variability of phasing tools. This variability is not reflected in overall summary metrics of phasing error. Figure 2b illustrates the different estimated haplotypes obtained by the six considered tools for a random individual within the same region. The example shows a contrast between the summary metrics and the resulting haplotypes. For example, EAGLE2 and BEAGLE both had a switch error of 5.4% (20 switches/372 heterozygous SNPs) in the same example, yet the estimations are different. Such examples motivate the development of metrics that may be more relevant to phased haplotype association analysis and that capture the error rate of the haplotype blocks used for downstream statistical analysis. Thus, we find that it is difficult to judge which estimation is better without considering the downstream application that will make use of the phase information.

### A consensus haplotype method improves phasing accuracy

The differences between the tools encouraged us to construct consensus haplotypes from the output of different tools. Due to its long runtime, MaCH was only examined on chromosome 17 and thus was not considered in any consensus combinations.

The results in Table 1 shows that no single tool obtained the best results for the three chromosomes. The minimum switch error is obtained by SHAPEIT2 for chromosome 1, BEAGLE for chromosome 6, and EAGLE2 for chromosome 17. These three tools were found to be consistently always substantially better than the remaining four tools, with fastPHASE demonstrating the highest switch error.

**Table 1:** Switch error (%) obtained by the tools when applied on chromosomes 1, 6, and 17

Approach	Tool	Chr1	Chr6	Chr17
Individual	BEAGLE	1.39	**1.12**	2.03
	SHAPEIT2	**1.33**	1.20	2.06
	EAGLE2	1.36	1.6	**1.90**
	HAPI-UR	2.14	1.81	3.00
	IMPUTE	2.83	2.46	4.30
	fastPHASE	4.10	3.43	5.32
	MaCH	-	-	4.10
Consensus	SHAPEIT2, EAGLE2 and BEAGLE	**1.14**	**0.98**	**1.68**
	SHAPEIT2, EAGLE2, BEAGLE, IMPUTE and HAPI-UR	1.2	1.04	1.76
	SHAPEIT2, EAGLE2 and HAPI-UR	1.24	1.08	1.79
	SHAPEIT2, BEAGLE and fastPHASE	1.37	1.14	2.06
	SHAPEIT2, EAGLE2, IMPUTE, fastPHASE and HAPI-UR	1.41	1.19	2.1
	EAGLE2, IMPUTE and HAPI-UR	1.48	1.27	2.16
	SHAPEIT2, fastPHASE and HAPI-UR	1.66	1.43	2.41
	IMPUTE, fastPHASE and HAPI-UR	2.16	1.82	3.19

Numbers in bold are the minimum error obtained according to each approach while underlined numbers are the minimum error obtained in all applied tests. MaCH was applied only on chromosome 17 due to the intensive performance time needed to phase other chromosomes. The tools are sorted according to the average switch error. The top section of the table lists the performance of the tools individually, while the bottom half lists the performance of the consensus estimators based on different combinations of phasing methods

Taking advantage of the independent phasing outputs between the individual tools, the best consensus, combining SHAPEIT2, EAGLE2 and BEAGLE, improves the accuracy for all datasets, with a 13% switch error improvement compared to the best single tool. However, if one of these tools is replaced with a less accurate tool (fastPHASE, IMPUTE and HAPI-UR), the consensus may provide worse results than one of the individual tools. Moreover, the addition of less accurate tools in consensus construction, as exemplified by the consensus using 5 tools, can also increase the overall error rate. Given the strong initial results for the consensus formed by SHAPEIT2, EAGLE2 and BEAGLE, we consider this approach in the rest of the tests reported in this study.

### Accuracy of haplotype blocks varies according to the block determination method

#### An evaluation of haplotypes obtained by a sliding window

**Fig. 3:**
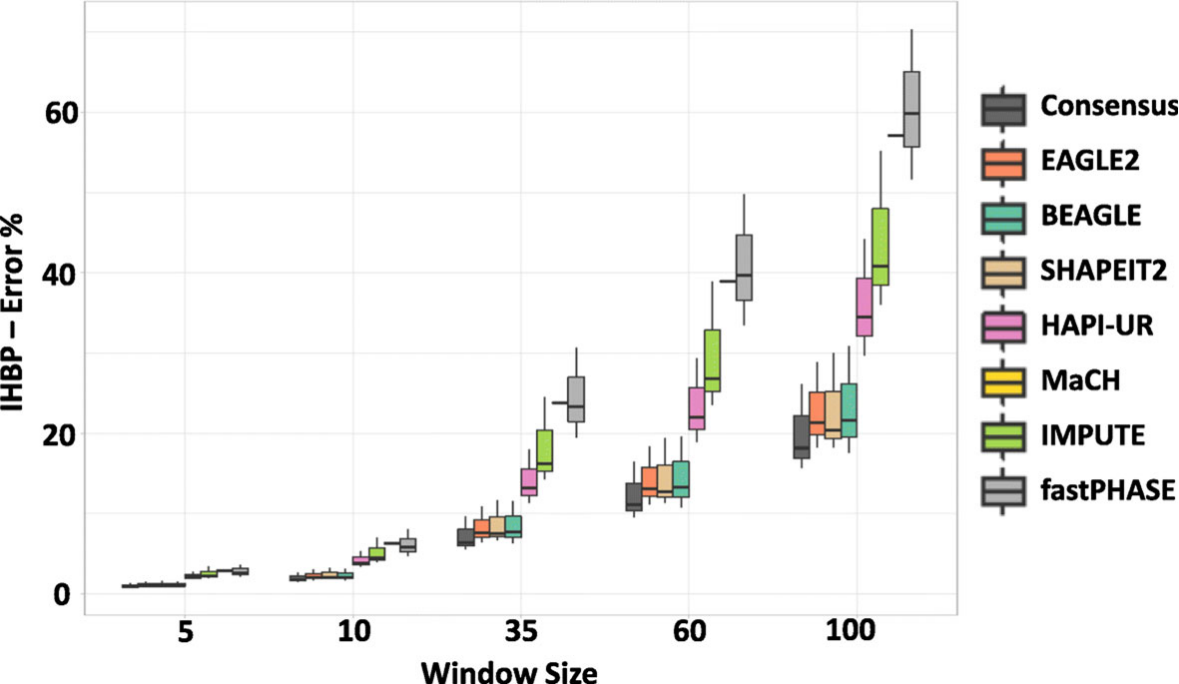
Impact of sliding window size on IHBP: Box plot summarizing the incorrect haplotype blocks percentage (IHBP) when applying different window sizes (5, 10, 35, 60, and 100 SNPs) to chromosomes 1, 6, and 17. The x-axis represents the window size, while y-axis represents shows IHBP. MaCH was only applied on chromosome 17 due to the extensive performance time required to phase chromosome 1, and 6, therefore its results are represented as a line

We investigated the incorrect haplotype block percentage (IHBP, see Evaluation Criteria in Methods) obtained by a sliding window applied on chromosomes 1, 6 and 17. Figure 3 shows the strong impact of the window size on IHBP for all tools, which varied from an average IHBP of 2% to more than 50% when the window length increased from 5 SNPs to 100 SNPs. The haplotypes obtained by the consensus approach had the minimum IHBP for all window sizes, while EAGLE2, SHAPEIT2, and BEAGLE clearly outperform the remaining phasing approaches. The difference of the average IHBP (for all datasets and windows) between the consensus haplotype and the best single tool is almost 10 times higher than the difference between the best two single tools. While we do not attempt to define an optimal window width for all cases, longer haplotype windows are always more likely to contain a phasing error compared to shorter windows as shown in Fig. 3. Moreover, Fig. 3 indicates that a long sliding window approach, as used in previous work [9], may have a high error rate, when a comparable sample of unrelated individuals is used.

Since the error rate of phasing tools is likely to vary dramatically given differences in recombination rates, heterogeneity and SNP density, we have also considered the relationship between the phasing error rate and the number of incorrect blocks for the seven haplotype estimation tools. We sample regions of 1400 contiguous SNPs 50 times from chromosome 17, this time categorizing them based on the length of their correctly phased runs, i.e. the average number of contiguous SNPs that are correctly phased in respect to each other.

**Fig. 4:**
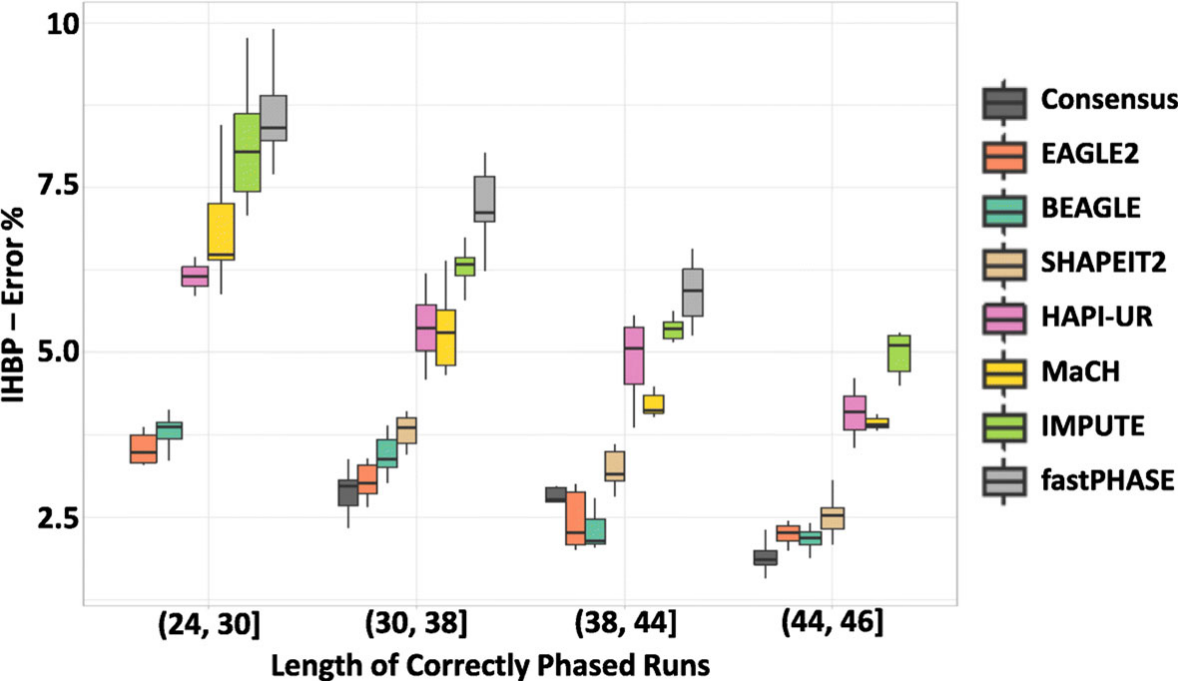
The relationship between the length of the correctly phased runs and IHBP: The incorrect haplotype blocks percentage (IHBP) was calculated for a fixed window size (10 SNPs) applied to 50 datasets from chromosome 17. The x-axis shows the correctly phased runs, binned according to the quartiles of the data. The y-axis shows IHBP. There is no bar for the consensus method in the range (24,30] as the length of the correctly phased runs always exceeds this range

Figure 4 illustrates how, for a fixed window size, the number of incorrect haplotype blocks increases for more difficult to phase regions, where phasing difficulty is measured using the length of correctly phased runs. Taking EAGLE2 as an example, we see that in regions that have longer correctly phased runs (44 to 64 consecutive correctly phased SNPs), only 2% of haplotype blocks contain errors. In contrast, in more difficult to phase regions (24 to 30 consecutive correctly phased SNPs), a median of 3% of blocks contain errors. While these results indicate that error rates between different regions of the same genome can vary by over 50%, we note that similar observations can be seen between regions on different chromosomes.

In these scenarios, the consensus method had the minimum IHBP for different window sizes, and different regions. Moreover, there is no result for the haplotype obtained by the consensus method when the length of the correctly phased runs is less than 30 SNPs. This observation demonstrates the efficacy of the consensus method as it was able to improve the accuracy and increase the length of the correctly phased runs to exceed this range. EAGLE2, BEAGLE, and SHAPEIT2 were the best tools individually.

#### An evaluation of haplotypes blocks obtained by lD

The phasing error rate of blocks defined based on LD, measured again as IHBP across chromosome 1, 6, 17, is summarized for the different haplotype estimation tools in Table 2. Consistent with results in the previous sections, the consensus haplotype caller had the minimum IHBP while SHAPEIT2, BEAGLE and EAGLE2 showed substantially less error than the remaining individual tools. Around 50% of the blocks incorrectly phased by HAPI-UR are caused by incorrect imputation of at least one missing SNP located in the block.

**Table 2:** Incorrect haplotype block percentage (%) obtained by the tools when applied on chromosomes 1, 6, and 17

Tool	Chr1	Chr6	Chr17
Consensus haplotype	**0.45**	**0.41**	**0.46**
BEAGLE	0.46	0.43	0.49
SHAPEIT2	0.47	0.42	0.51
EAGLE2	0.48	0.44	0.48
HAPI-UR	1.26	1.27	1.25
IMPUTE	1.17	1.18	1.47
fastPHASE	0.54	0.5	0.58
MaCH	-	-	0.54

Numbers in bold are the minimum error obtained by any approach while underlined numbers are the minimum error for a single tool. MaCH was excluded from the tests applied on chromosome 1, and 6 due to extensive performance time

**Fig. 5:**
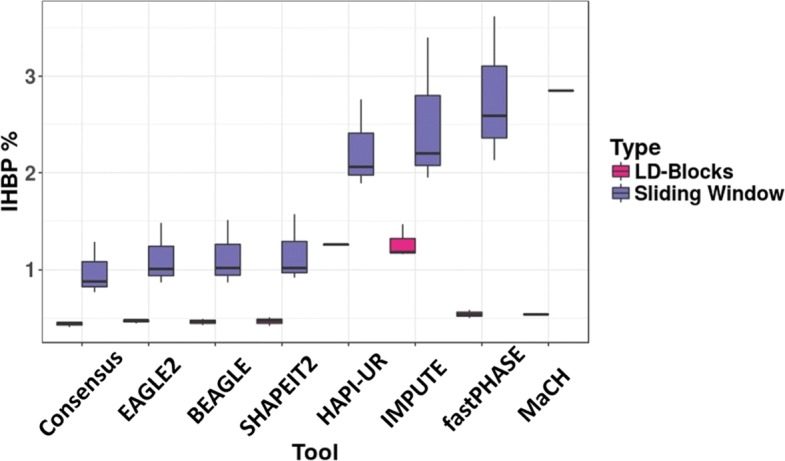
Comparison of haplotype block determination approaches. Boxplot showing the incorrect haplotype block percentage calculated for the blocks obtained by sliding window (5 SNPs width) or using an LD-based approach on the same dataset for each phasing method

In order to compare the error rate for sliding window and LD based block determination approaches, we used a sliding window with a width of 5 SNPs, equal to the mean of SNP count with blocks determined by LD. Both approaches were applied on chromosome 1, 6, and 17, and the incorrect haplotype block percentage was calculated for each tool and summarized in Fig. 5. We see that the error rate was halved in many cases when using an LD block approach (with a variable length from 2 SNPs to 34) compared to that of a sliding window. However, the number of blocks to be evaluated is greatly altered between the approaches, with a median number of blocks 6056 (interquartile range (IQR): 4390–6546) vs 31,729 (IQR: 22,269–34,327) for the LD-based and 5-SNP sliding window approach, respectively. These results highlight the trade-off between accuracy and comprehensiveness that to need be made when selecting a block determination methods to use in phased haplotype analysis.

### Impact of including surrounding regions on haplotype estimation

Haplotype studies interested in specific regions or genes [9, 20, 21], often phase only these particular regions rather than the whole chromosome. Therefore, we investigated the error rates of specific haplotype blocks when phasing either the entire genome or just the regions containing blocks of interest using SHAPEIT2, EAGLE2 and HAPI-UR.

**Fig. 6:**
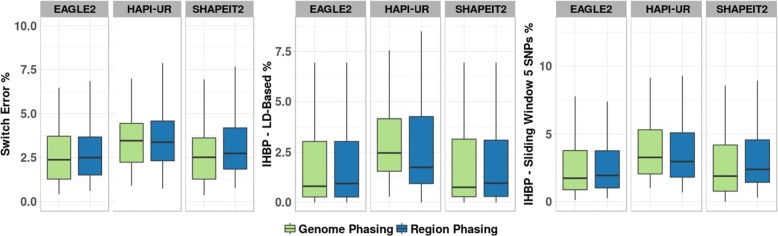
Evaluation Tests when including and excluding surrounding regions. Boxplots summarize switch error and IHBP with blocks determined using LD-based and sliding window (5 SNP) when phasing using SHAPEIT2, EAGLE2, or HAPI-UR is applied on the same regions in two different scenarios: one with including surrounding regions (green), and when only including target region (blue)

Figure 6 shows that there are only small difference in accuracy regardless of whether phasing is performed on entire chromosomes or only selected regions. The error rate obtained by SHAPEIT2 and EAGLE2 was reduced for all metrics when phasing the whole genome followed by extraction of the regions of interest; however, the magnitude of improvement was small. When including surrounding regions, the median of sliding window-IHBP was reduced from 2.4 to 1.0% when applying SHAPEIT2 and from 1.94 to 1.73% when applying EAGLE2. HAPI-UR had different behaviour, achieving better results when phasing the short regions, increasing the IHBP from 2.96 to 3.27% when using a sliding window approach. However, given the poor performance of HAPI-UR on other evaluations in this work, it is unclear whether this result is likely to generalize to other tools. These results indicate that phasing the entire genome is likely to lead to improved results compared to phasing only specific regions, but the improvement may not warrant the additional computation time.

### Tool stability

We observed that the outputs of many of the phasing methods evaluated changed across multiple runs when the input remained the same, with this instability having potential to affect the replication of downstream analysis. While this observation was not made for EAGLE2, similar instability could be observed by permuting the order of individuals in the dataset being analysed. To explore this instability, we conducted a stability test for five tools.

**Fig. 7:**
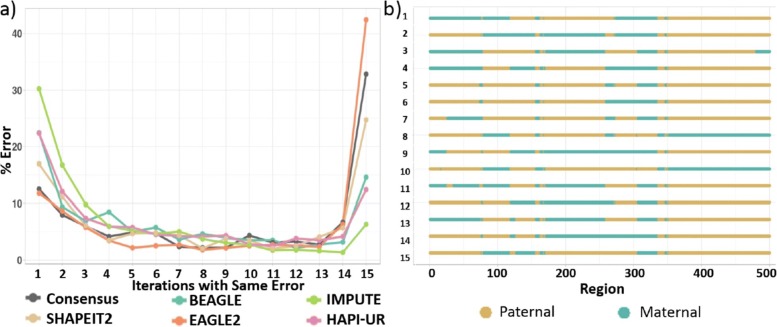
Stability of switch error. **a** The percentage of errors that are consistent across different runs of the same phasing method and same data. For example, the point (2, 9) in BEAGLE plot means that 9% of the errors reported by BEAGLE within 15 iterations occurred in the same location only in two iterations. **b** 15 different estimations for the same random individual (around 500 SNPs within the region chr1:212540742-224862598) obtained by SHAPEIT2 tool

Figure 7a illustrates the consistency of identifying a SNP location as a switch error across 15 runs. This distribution shows that the majority of the errors were either unique for each run, or were common across the 15 runs. The minimum error location variation was obtained by EAGLE2 followed closely by the consensus approach. The large proportion of errors unique to each run indicate that there is a substantial amount of variation between different runs of the same tool.

Figure 7b illustrates the differences between the estimations of SHAPEIT2, consistently one of the most accurate phasing tools, across the 15 iterations. This plot shows how the characteristics of the data influence the accuracy of the haplotype estimation. Here, we find that the estimation for the region from [350 to 500] was similar across most of the 15 iterations, while the results vary substantially for the region at [0 to 350]. Such variation is highly likely to affect downstream analysis and represents a significant issue for haplotype association analysis.

**Fig. 8:**
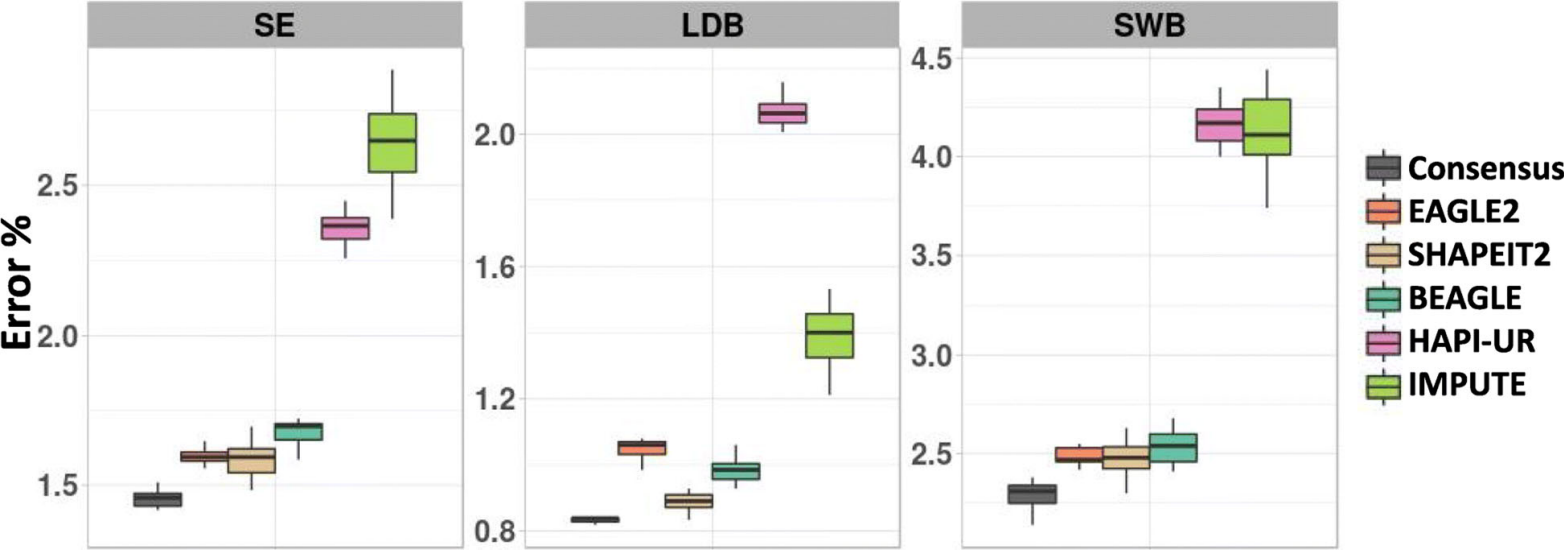
Impact of phasing method instability on error rates. Boxplot summarizing the variation of the obtained errors when applying the tools on the same dataset 15 times. The plots are, from left to right: switch error (SE), IHBP calculated for blocks determined according to the LD (LDB), and IHBP calculated for blocks determined by sliding window with a width of 10 SNPs (SWB)

While Fig. 7 highlights the variability of the error locations, we can also examine how the instability of the phasing tools impacts the error rates of the haplotype blocks determined via sliding window and LD. Figure 8 illustrates the variance of errors obtained by each tool across multiple runs and highlights that there is substantial variability across different applications of the same tool. For instance, SHAPEIT2 shows switch error varying around 15% difference between the best and worst performing iteration of the tool. For all metrics, we again see the consensus approach obtains the best accuracy (in line with previous results). However, its variability is similar to BEAGLE and SHAPEIT2, as it is based on stochastic tools.

## Discussion

Improving the accuracy of phased haplotypes and understanding the impact of block determination methods on the accuracy of the haplotype blocks is critical to conducting accurate and replicable haplotype association analysis. This is the first study to evaluate phasing tools and their relationship with two standard methods for determining haplotype blocks with a focus on phasing accuracy at block scale. Our evaluations showed that three tools, SHAPEIT2, EAGLE2, and BEAGLE, consistently obtained the best results in all applied tests, with performance between these tools varying depending on the scenario and data under evaluation. This observation is in line with other studies that evaluated overall switch error [11, 19]. A consensus approach built from these three tools led to further improvements in switch error and incorrect haplotype block percentage. We demonstrated the trade-offs between phasing accuracy, block length and block count that are inherent between LD and sliding window based methods for block determination. Finally, we examined the stability of these tools and demonstrated that there is a large variability in outputs for most tools between runs.

### Ensemble approach improves haplotype estimation for all considered scenarios

While most previous evaluation of phasing tools focuses on overall switch error rates, our analysis compared the error profile from the different tools (Fig. 2). The comparison revealed that the switch error from different tools occurs in different locations, encouraging us to construct a consensus haplotype from the output of multiple phasing methods. A key outcome of this work is the robust phasing accuracy achieved by the consensus approach constructed from SHAPEIT2, EAGLE2 and BEAGLE across all metrics for all applied tests. This approach achieves a 13% improvement in switch error compared to the best individual tool and indicates a strong improvement compared to previous methodological phasing advances [22–24]. As expected, the performance of the consensus is influenced by the individual tools used in its construction, with less accurate tools lowering performance but remaining more accurate than any individual tool.

While the consensus of SHAPEIT2, BEAGLE and EAGLE2 gives the lowest error rates in all scenarios examined in this paper, the results in Table 1 show that there is no guarantee that such an ensemble approach will outperform its constituent tools individually, with the inclusion of fastPHASE in particular dragging down performance of any consensus it was included in. However, if the tools included in the ensemble are comparable in terms of accuracy, we observe that the resulting consensus is likely to lead to improvements in performance and robustness.

The downsides of the consensus caller approach are the increases in runtime, due to multiple passes over the same data. Hence, the tools may not scale effectively to large datasets. In this study, BEAGLE was the bottleneck of the consensus, taking one month to phase chromosome 1 for 12,008 individuals, while EAGLE2 and SHAPEIT2 accomplished the phasing task within a week, albeit using only a single thread of an AMD Opteron 6200. However, given that running multiple tools can be trivially parallelized and that haplotype phasing only needs to be run once for downstream analysis, the gain of the accuracy obtained from the consensus approach may justify the additional run time required.

### Trade-offs in block determination methods

The differences between the choice of LD and sliding window approaches for block determination in haplotype association analysis have long been recognized [17]. The former tends to produce fewer blocks whose length adapts to recombination properties of the genomic region under consideration, while the latter produces many, typically longer blocks with the same length across the genome. In this work, we have explored the impact that these trade-offs have on phasing error, focusing on IHBP to evaluate the proportion of blocks that are incorrectly phased. This metric more clearly elucidates the likely impact of phasing errors and block determination on the downstream application of statistical association analysis. When using a fixed size sliding window, our results showed a significant impact of the window size on the error rate of the haplotype blocks (Fig. 3), with error rates varying significantly depending on the region being phased (Fig. 4). The error rate variation, influenced by factors such as the recombination rate and the density of SNPs, demonstrates that a fixed size sliding window is likely to lead to many haplotypes that contain errors. This is highly likely to have a strong impact on the false negative and false positive rates of downstream analysis and is likely to place some limitations on the replicability of results.

In order to minimize the error rate within the haplotypes obtained by a sliding window, the width of the window should suit the length of the correctly phased runs. As there is no optimal way to determine the best window width, one solution may be an adaptive window size whereby the size of the window is adjusted according to the characteristics of the scanned region (e.g. is smaller if recombination rates are high or SNP density is low). Such an approach has been explored in the literature [25] and our results lend further support for such an approach. Alternatively, an LD-block approach may be used as this inherently adapts to the properties of the data, albeit with a number of settings that need to be defined by the user. We found that such an approach led to a strong reduction in switch error rates and the number of defined blocks that contain errors, at a cost of producing fewer, shorter block segments. This may be detrimental if the variation of a single region is needlessly broken into multiple independent blocks. We note it may be possible to use the consensus approach to determine block boundaries guided by whether individual methods are consistent at a given position or even to derive probabilistic block boundaries by treating the consensus caller as an ensemble predictor [26]. Combining this with probabilistic tests for haplotype association analysis [3, 14] may further reduce type 1 error rates in downstream analysis.

### Tool stability

All algorithms for phase estimation considered in this work are based on Hidden Markov models which are often trained using a stochastic algorithm, which may explain why most phasing methods explored have different outputs across multiple runs when the input and parameters remains constant. Only EAGLE2 produces stable outputs by default. Although the variation in the error rate for most phasing methods was small across different iterations, the switches or the boundaries of correctly phased runs were affected significantly. The majority of the switches in the estimated haplotypes were either common across 15 iterations or unique to each iteration. We note that while some of the tools allow for a random seed to be passed in as a parameter, the default setting for the methods uses random initialization and that such seed parameters typically have no effect if the tools are run in a multi-threaded mode. Moreover, small changes in the input data, such as reordering samples, also appears to change the phasing output. As such, we believe that the instability observed in this study reflects the way that these phasing tools are being used in most practical applications.

The consensus haplotypes not only improved the accuracy but also had very stable results, rivalling those of EAGLE2. This instability of error location can be seen as similar to the difference in error location shown in Fig. 1b). As such, it may also be possible to exploit the instability of the tools to construct a consensus haplotype from the same tool applied several times on the same dataset. In this way it may be possible to yield the robust results from the consensus, while only running a single efficient tool.

An important question that arises here is whether the instability of the tools has a significant influence on detected results. Given that we observe relatively high error variation across the same data, one could imagine that the different tools may vary even more dramatically across different datasets. This is likely will not affect EAGLE2, the only stable tool, as its stability is based on the seeding conditions for a given set of input data and when that data changes, variation may occur. This variability in output is likely to be a key limitation on the replicability of haplotype association analysis. Future research could explore how the stochasticity of phasing algorithms could be exploited to reduce phasing error rates.

### The impact of including surrounding regions on phasing

Haplotype studies interested in specific regions or blocks, such as those focusing on genes only [20, 21] or the replication of association analysis after finding genomic regions with significant associations [9], often phase only these particular regions rather than the whole genome. However, the choice to only focus on select genomic regions and phase their blocks, as opposed to phasing the genome and then extracting blocks, may affect the quality of phasing. We have found that SHAPEIT2 and EAGLE2 perform better when including surrounding regions while HAPI-UR performed better when phasing only the region of interest. While EAGLE2 is the only stable tool according to the stability tests (using default parameters), it obtained different results for the same region when including or excluding surrounding regions. These results indicate that phasing the entire genome is likely to lead to improved results compared to phasing only specific regions. However, the improvement may not warrant the additional needed computation time.

### Limitations of the study

Our primary goal was to evaluate the interaction between the choice of phasing tool and the block determination algorithm and the impact these had on phasing error rates. In particular, the analysis conducted in this work did not explicitly explore the impact that these had on downstream haplotype analysis, given the large amount of variability that this could entail (different choices of statistical tests, different assumptions of genetic architecture, etc.). In order to reduce the number of analyses conducted, we have also limited the parameter options of both the phasing tools selected and the block determination algorithms to their default parameters unless otherwise explicitly stated. Finally, all error rates produced here were on a small subset of individuals for whom phase information was available (n=39). While this sample size is comparable with previous evaluations [11, 27], increasing the number of individuals for whom resolved phased information is available would help further refine these results and the differences between evaluated tools.

## Conclusions

This study reports on the interaction between the choice of phasing tool and the block determination algorithm, with critical implications for the application of phased haplotype blocks such as haplotype association analysis. We provide a comprehensive evaluation of seven different haplotype phasing tools (fastPHASE, BEAGLE, IMPUTE, MaCH, SHAPEIT2, HAPI-UR, and EAGLE2). We further introduce a consensus haplotype estimator based on combining output from multiple phasing tools, that achieved the lowest error rates across all scenarios considered.

The work provides guidance and evidence for the key constituent methods of haplotype analysis at block scale by showing the positive and negative consequences of each choice independently and when used together, as well as highlighting the possibility of tool instability. The insights provided by this work should inform future haplotype-based analyses as well as drive methodological research into phasing tools.

## Methods

### Datasets and preparation

Real haplotype data for a population is not readily available for comprehensive evaluation. One common approach to determine phasing data is to use data from trios to resolve child haplotypes based on the sequence of the parents [11, 16]. In this work, we make use of trios obtained from HapMap project [28] using Utah Residents (CEPH) with Northern and Western European Ancestry (CEU). This dataset contains 19 unrelated individuals and 39 complete families consisting of father, mother and child (117 individuals, of these 39 children were included while 78 parents were excluded for our study). Parent data were used to resolve child haplotypes but were otherwise excluded as our focus is on phasing unrelated individuals.

For phased children, 35% of SNPs were heterozygous, 0.3% were missing (unknown whether heterozygous or homozygous) and 0.08% were Mendelian errors (inconsistent alleles among trios). Using the parent information, we could resolve 80% of the child heterozygous SNPs, and 40% of the missing SNPs. Heterozygous SNPs for each child were resolved deterministically using the parent information via “phasing by transmission”. A child’s heterozygous and missing SNPs are resolved when at least one of its parents has a homozygous SNP in the same genomic locus. Haplotypes for the 39 children were extracted from chromosomes 1 (36,923 SNPs), 6 (31,727 SNPs), and 17 (12,807 SNPs). The restriction to three chromosomes was related to the high runtime needed by some of the tools under evaluation.

The genotype data for the 39 CEU children and 19 unrelated individuals were combined with 11,950 individuals from coeliac disease dataset (EGA accession: EGAS00000000057). While the additional genomic data does not have resolved phase information, the large number of samples has been shown to improve the accuracy of haplotype estimation [16] and enables us to emulate the performance of phasing tools in a real-life scenario. The coeliac disease dataset contains 11,950 individuals (cases and controls) and 528,969 SNPs (Illumina Hap 550). Details on the collection, and quality control procedure applied on the dataset is described previously [29].

Evaluation and error metrics calculation were computed only from the 39 children with partially known haplotypes, while the haplotype estimation tools were applied to the entire dataset of 12,008 individuals, in order to maximise phasing accuracy.

### Haplotype estimation methods

The seven haplotype estimation tools evaluated in this study are summarized in Table 3. These tools are all based on the probabilistic Hidden Markov Model (HMM) framework of Li and Stephens [30]. However, a direct application of the Li and Stephens approach scales linearly with the number of individuals and the number of loci and quadratically with the number of possible haplotypes, limiting its direct applicability to large datasets. Given this, phasing tools have introduced heuristics to reduce the computational cost of phasing while trying to limit the impact on phasing accuracy. A brief summary of the heuristic used by each tool is given in Table 3. All the tools were applied using their default parameters. Genetic maps, which contain information about recombination rates across the genome, were used as an additional parameter to most tools (all except fastPHASE and MaCH) with EAGLE2 taking a specific format^1^ while all other tools used a PLINK format^2^. All genetic maps were made with respect to the GRCh36 reference genome (genome build hg18).

**Table 3:** Population-based haplotype estimation tools used in this study

Tool-Version	Year	Heuristic to reduce haplotype search space
fastPHASE - 1.4.8	2006	Uses a haplotype-clustering model, where the set of all possible haplotypes are clustered into a small fixed number of “ancestral” haplotypes [31].
BEAGLE - 4.1	2017	Uses a haplotype-clustering model with a variable number of clusters, depending on the region under consideration [24].
IMPUTE - 2.3.2	2009	Subsamples possible haplotypes that are similar to those of the currently estimated haplotype of an individual [32].
MaCH - 1.0.18.c	2010	Subsamples possible haplotypes from the set of all possibilities randomly at each iteration [33].
SHAPEIT2 - v2.r837	2012	Breaks the chromosome into small windows of a few SNPs, estimates the phase of each window using a method similar to IMPUTEv2 and then estimates transitions between windows [23].
HAPI-UR - v1.01	2012	Breaks chromosome into small windows, that are initially very short but iteratively grow to a user defined size, enabling modelling of longer segments at once [34].
EAGLE2 - v2.3.5	2016	When no reference panel is provided (the scenario in this study), EAGLE2 applies long range phasing (EAGLE1 [27]) then efficiently represents all haplotypes such that beam search can be applied to evaluate only the most promising phase paths [22].

### Consensus haplotype construction

We constructed a consensus haplotype of the estimations obtained by several combinations of three and five tools. The consensus haplotype is assembled as follows:

1. The haplotypes of the tools are aligned to each other (one copy of the estimated haplotype pair from each tool). The haplotypes should have the same allele at the first heterozygous SNP.
2. Scan the haplotypes SNP by SNP, and for each SNP, vote for the alleles and choose the final one (for the consensus estimator) according to the majority.
3. If the allele at the scanned SNP for a tool doesn’t agree with the final allele (based on the majority of tools), switch the copies of the chromosome pair for the remaining SNPs for this specific tool.

### Evaluation criteria

The standard metric to assess the accuracy of haplotype estimation is switch error, the number of switches in the estimated haplotype divided by all possible switches (heterozygous SNPs count) [15, 16]. Most studies measure switch error as it reflects the accuracy with respect to the neighbouring SNPs [24]. Switch error is formally defined as:

1 $$ SE = \frac{\sum_{i=1}^{n} \frac{switches}{H - 1}}{n}  $$

where *switches* is the number of the incorrectly phased heterozygous SNPs in comparison to their predecessor SNPs (SNPs in the previous genomic position), *H* is individual’s heterozygous SNPs count, and *n* is the individuals count in the dataset.

In this study, we focus on the accuracy of phased haplotypes with respect to the block determination approach. Therefore, we introduce a new accuracy metric termed Incorrect Haplotype Block Percentage (IHBP) in order to calculate the error rate within blocks determined either by sliding window, LD between SNPs or any other approach. The formula for IHBP is defined as:

2 $$ IHBP = \frac{IB}{B}  $$

where *IB* is the count of the incorrectly phased haplotype blocks and *B* is the count of all haplotype blocks. All unambiguous blocks, i.e. blocks that contain only homozygous or one heterozygous SNPs, were also excluded. A haplotype pair within a block is considered correctly phased if all heterozygous and missing SNPs are phased correctly with respect to each other. In other words, one copy of the pair is identical to the paternal haplotype, and the second copy is identical to the maternal haplotype within the same block (though we do not explicitly identify the origin of each phased sequence).

When calculating error metrics, all unresolved SNPs (child’s heterozygous SNPs when both parents have heterozygous SNPs in the same loci) and Mendelian errors were excluded from the evaluation. Missing SNPs that were resolved using family information were included in the evaluation as all haplotype estimation tools used in this study implicitly impute missing SNPs.

An alternative metric of phase accuracy is length of correctly phased runs, defined as

3 $$ LCPR = \frac{\sum_{i=1}^{n} {LR}_{i}}{n}  $$

where *LR*_*i*_ is the length of the correctly phased run *i*, and *n* is the number of the correctly phased runs within a region. A correctly phased run refers to contiguous sets of correctly phased heterozygous SNPs with respect to each other. The length of the correctly phased runs within each region was calculated as the mean of the number of heterozygous SNPs within each run.

### Haplotype blocks determination via sliding window

The sliding window scans the whole chromosome in this work shifting one SNP step at a time, treating each window as a haplotype block. The possible haplotype blocks within a sequence of *m* SNPs are *m*−*w*+1 blocks, where *w* is the window size. Phasing accuracy of blocks determined by a sliding window with five different random sizes (5, 10, 35, 60, and 100 SNPs) and one SNP step was investigated in this study with respect to chromosomes 1, 6 and 17.

### Linkage disequilibrium (LD) based haplotype blocks determination

In this study, LD-based blocks were determined which implements the confidence interval (CI) algorithm [35] as implemented by PLINK (v1.90b4.4) [36]. The algorithm requires a number of heuristic thresholds to be set and, in line with previous haplotype studies [12], we have made use of the following default parameters: SNP pairs are considered if they are within 200 kilobases (kb) of each other. SNPs with minor allele frequency (MAF) <0.05 were excluded. SNP pairs are considered highly correlated (belong to the same block) if the bottom of the 90% *D*^′^ confidence interval is >0.70, and the top of the confidence interval is >0.98.

To examine the error rate of the resulting blocks, we consider the IHBP from ∼4800 LD blocks derived from chromosome 1, 6, and 17 for the 39 CEU children. This analysis excludes haplotype blocks which contained fewer than 2 heterozygous SNPs (i.e. which blocks which needed no phasing). The average SNP count within these blocks is 4.3 SNPs (minimum 2 SNPs and maximum 34 SNPs). The average length of these blocks is 17.5 Kb (ranging from 0.005–200kB).

### Analysis of including surrounding regions on phasing particular regions

Two scenarios were applied for this investigation:

1. Phasing then block determination: Phasing methods were applied on the whole chromosome, then the estimated haplotypes were extracted to obtain the targeted region.
2. Block determination then phasing: Phasing methods were applied on specific regions without including any other neighbouring parts of the chromosome.

60 datasets were constructed by randomly selecting 150 or 250 contiguous SNPs from chromosome 1, 6, and 17 from 6000 individuals (including all 39 CEU children). Short regions were chosen as excluding or including surrounding regions will affect phasing accuracy in the boundary of the region of interest, and also for execution time issues as this evaluation was applied to 60 different regions. Only the fastest tools (SHAPEIT2, EAGLE2, and HAPI-UR) were used for this test. We compared the error rate within the results in both scenarios for all accuracy metrics. Both Switch Error and IHBP were calculated for each scenario, with IHBP calculated for blocks determined by 5-SNP sliding windows and based on LD.

### Stability testing

The tools were applied to a randomly selected region of 2000 heterozygous SNPs (chr1:212540742-224862598) for 4000 individuals. The size of the region was limited to this size due to computational constraints, based on the time needed to execute 5 tools 15 times. MaCH and fastPHASE were not used in this comparison due to excessively slow performance. EAGLE2 was stable when using its default parameters. Therefore, we created 15 different datasets of the same region but with shuffling the individuals randomly for each of them. Shuffling the individuals made EAGLE2 unstable and allowed the assessment of its behaviour. Three error metrics were calculated: switch error and IHBP based on either LD-based or 10-SNP sliding windows.

## Abbreviations

CEPH: Centre d’Etude du Polymorphism Humain families reference panel
CEU: Utah Residents from the CEPH collection with Northern and Western European Ancestry
HAPI-UR: HAPlotype Inference for UnRelated samples
HMM: Hidden Markov Model
IHBP: Incorrect haplotype block percentage
IQR: Interquartile range
LCPR: Length of correctly phased runs
LD: Linkage disequilibrium
LDB: IHBP calculated for blocks determined according to the LD
MAF: Minor allele frequency
SE: Switch error
SHAPEIT: Segmented HAPlotype Estimation and Imputation Tool
SNP: Single-nucleotide polymorphism
SWB: IHBP calculated for blocks determined by sliding window

## Footnotes

1
https://data.broadinstitute.org/alkesgroup/Eagle/downloads/

2
http://bochet.gcc.biostat.washington.edu/beagle/genetic_maps/
